# A survey of decision-making and planning methods for self-driving vehicles

**DOI:** 10.3389/fnbot.2025.1451923

**Published:** 2025-02-18

**Authors:** Jun Hu, Yuefeng Wang, Shuai Cheng, Jinghan Xu, Ningjia Wang, Bingjie Fu, Zuotao Ning, Jingyao Li, Hualin Chen, Chaolu Feng, Yin Zhang

**Affiliations:** ^1^School of Software, Shenyang University of Technology, Shenyang, China; ^2^Neusoft Reach Automotive Technology (Shenyang) Co., Ltd., Shenyang, China; ^3^School of Electronics and Information Engineering, Liaoning University of Technology, Jinzhou, China; ^4^School of Computer Science and Engineering, Northeastern University, Shenyang, China; ^5^Key Laboratory of Intelligent Computing in Medical Image, Ministry of Education, Shenyang, China

**Keywords:** autonomous driving technology, decision-making and planning algorithms, hybrid models, data-driven, knowledge-driven

## Abstract

Autonomous driving technology has garnered significant attention due to its potential to revolutionize transportation through advanced robotic systems. Despite optimistic projections for commercial deployment, the development of sophisticated autonomous driving systems remains largely experimental, with the effectiveness of neurorobotics-based decision-making and planning algorithms being crucial for success. This paper delivers a comprehensive review of decision-making and planning algorithms in autonomous driving, covering both knowledge-driven and data-driven approaches. For knowledge-driven methods, this paper explores independent decision-making systems, including rule based, state transition based, game-theory based methods and independent planing systems including search based, sampling based, and optimization based methods. For data-driven methods, it provides a detailed analysis of machine learning paradigms such as imitation learning, reinforcement learning, and inverse reinforcement learning. Furthermore, the paper discusses hybrid models that amalgamate the strengths of both data-driven and knowledge-driven approaches, offering insights into their implementation and challenges. By evaluating experimental platforms, this paper guides the selection of appropriate testing and validation strategies. Through comparative analysis, this paper elucidates the advantages and disadvantages of each method, facilitating the design of more robust autonomous driving systems. Finally, this paper addresses current challenges and offers a perspective on future developments in this rapidly evolving field.

## 1 Introduction

### 1.1 Background

Autonomous driving technology exemplifies a crucial application of robotics theories and techniques, aiming to ensure safe and efficient self-driving in real-world traffic environments (Zhang et al., 2024). Within autonomous driving systems, decision-making and planning algorithms are pivotal, tasked with generating driving behaviors and planning trajectories. Broadly autonomous driving encompasses two phases: behavior decision-making and motion planning. Behavior decision-making addresses responses to temporary events, such as abnormal driving behaviors of other vehicles, sudden pedestrian crossings, and emergency vehicle avoidance (Xu et al., 2024; Wang T. et al., 2024). At this phase, the decision system must exhibit high adaptability and predictive capability for potential future scenarios, allowing for quick adjustments like lane changes, acceleration, or deceleration based on real-time conditions (Yang et al., 2024; Feng et al., 2024). Motion planning delves into a more granular aspect of autonomous driving, generating detailed trajectories based on the current vehicle state and behavior decision outputs (Li Z. et al., 2023). The motion planning ensures the smoothness and comfort of the vehicle's trajectory while adhering to dynamic constraints such as speed and acceleration. Given the significant challenges associated with achieving safe and flexible interactions, behavior decision-making and motion planning have become critical focal points in autonomous driving research, which is also the primary subject of this paper.

The decision-making and motion planning algorithms for autonomous vehicles integrate theories from multiple disciplines, including machine learning, pattern recognition, intelligent optimization, and nonlinear control (Lu et al., 2024). Deep learning techniques effectively improve the ability to encode model features (Bidwe et al., 2022). These technologies provide the foundation for safe interactions between autonomous vehicles and other road users on public roads. Furthermore, decision-making and planning algorithms must consider ethical and legal responsibilities, ensuring adherence to socially accepted moral standards and compliance with traffic regulations during emergencies (Zheng et al., 2024; Gao et al., 2024). Current research on decision-making and planning algorithms focuses on improving robustness, enhancing stability and safety in unforeseen situations, and increasing predictive accuracy of the surrounding environment and other traffic participants (Wen et al., 2023; Wang W. et al., 2023; Zhai et al., 2023). Additionally, efforts are being made to reduce computational resource consumption and improve algorithmic efficiency to achieve rapid responses under resource-constrained conditions.

Aiming at the aforementioned research objectives, the researchers primarily employ knowledge-driven and data-driven approaches to construct decision-making and motion planning systems. The knowledge-driven approach simulates human decision-making processes through the encoding of expert knowledge and logical rules. By integrating information such as road characteristics, traffic regulations, and historical behavior data, these approaches can search for the optimal driving path or optimize for a specific objective function, thereby achieving safe and efficient driving strategies (Jia et al., 2023; Chen L. et al., 2023; Aoki et al., 2023). Concurrently, data-driven approaches have emerged prominently propelled by advancements in machine learning and statistical analysis. Unlike knowledge-driven methods, data-driven approaches do not necessitate pre-defined explicit rules. They enhance decision accuracy and adaptability by training and optimizing decision models using vast amounts of real driving data. Particularly in complex and dynamic traffic environments, data-driven strategies effectively learn and emulate human driver behaviors and decision-making processes (Wang T. H. et al., 2023). The application of data-driven technologies also significantly enhances their generalizability across different environments. However, solely relying on data-driven methods has its limitations; these methods typically require large volumes of labeled data for training and often have poor interpretability, making it challenging to ensure consistent and safe decisions.

On the other hand in industry, the deployment of autonomous vehicles (AVs) is incrementally expanding, particularly within the commercial sector. The available AVs on the market primarily employ several key decision-making and planning methodologies. These include rule-based systems, state transition models such as Markov Decision Processes (MDPs) and Partially Observable Markov Decision Processes (POMDPs), as well as game-theoretic approaches. These vehicles utilize sophisticated algorithms to process road environments and vehicle states, optimizing state transitions to make the best possible decisions.

The deployment of AVs is being tested and operated in specific geographic areas and under certain traffic conditions. Waymo and Tesla stand out as prominent examples. Waymo offers autonomous taxi services in Phoenix, Arizona, and is expanding its service reach. Reports indicate that Waymo plans to extend its services to Miami, Florida, in an effort to gain an edge in the intensifying competitive market. Moreover, Waymo has established a partnership with the automotive financing company Moove, which will manage Waymo's fleet operations in Phoenix, including maintenance of the autonomous taxis and management of charging infrastructure. Currently, Waymo has deployed approximately 200 autonomous vehicles in Phoenix. Tesla, on the other hand, collects data through its fleet learning program to improve its autonomous driving systems. While Tesla's Autopilot and Full Self-Driving (FSD) systems have made significant advancements in autonomous technology, there is still a considerable gap before achieving true Level 4 (L4) autonomous driving capabilities. Tesla employs an end-to-end (E2E) deep learning strategy, integrating neural networks and reinforcement learning in an attempt to enhance the intelligence level of autonomous driving. Tesla's Robotaxi technology faces challenges, including safety and reliability issues, regulatory and licensing hurdles, and market acceptance and operational challenges. These deployments demonstrate the applicability and challenges of autonomous technology under real-world conditions and highlight how industry leaders are testing and optimizing their technologies in specific geographic and traffic settings. As technology matures and regulatory environments adapt, it is anticipated that the deployment of AVs will become more widespread and in-depth.

Despite the immense potential of AVs, they still face certain limitations in decision-making and planning. These include interactions with human drivers under mixed traffic conditions, responses to unexpected situations, and adaptability within complex traffic environments. Additionally, AV decision-making and planning systems must consider ethical and legal responsibilities, ensuring adherence to socially accepted moral standards and compliance with traffic regulations during emergencies.

Neurorobotic approaches, which combine neural networks and robotics, offer new possibilities for AV decision-making and planning. These methods can improve the accuracy and adaptability of decision-making by learning from and optimizing decision models with extensive real-world driving data. Particularly in complex and dynamic traffic environments, data-driven strategies effectively learn and emulate human driver behaviors and decision-making processes, significantly enhancing generalizability across different environments. However, relying solely on data-driven methods has its limitations; these methods typically require large volumes of labeled data for training and often have poor interpretability, making it challenging to ensure consistent and safe decisions.

Thus, an increasing number of researchers are attempting to combine knowledge-driven and data-driven methods to complement each other. In this paper, we refer to these combined methods as hybrid methods. Hybrid methods harness the advantages of both approaches: the data-driven component improves the system's adaptability to complex environments and the accuracy of predictions by extracting patterns and behaviors from extensive driving data; meanwhile, the knowledge-driven component ensures decisions comply with traffic regulations and safety standards, providing systematic constraints and guidance within a well-defined framework. This combination allows for more flexible, robust, and interpretable decision planning (Singh, 2023). Although hybrid methods aim to integrate the strengths of knowledge-driven and data-driven approaches, they also present certain limitations and potential challenges in development. The design and implementation of hybrid methods are complex, requiring precise integration of two fundamentally different techniques. This not only demands strong theoretical knowledge from algorithm designers but also necessitates continuous tuning and optimization in practice to achieve optimal performance. By thoroughly discussing and comparing these algorithms, we aim to understand their respective advantages and limitations and explore effective ways to integrate these methods to tackle complex decision-making and motion planning problems in autonomous driving.

### 1.2 Paper structure

This review provides a comprehensive overview of decision-making and planning technologies in autonomous driving systems. The following sections detail the research progress in each aspect.

Introduction: The introduction reviews the essential role of decision-making and planning in autonomous driving systems, outlining the historical applications and unique advantages and limitations of knowledge-driven, data-driven, and hybrid methods. It provides a detailed comparative analysis of these methods, discusses their effectiveness in various scenarios, and summarizes the article's structure to offer a comprehensive understanding of the advancements in the field.

Knowledge-driven decision and planning methods: This section explores knowledge-driven decision-making and planning methods, focusing on the decision process and path planning process. It covers the framework of rule based systems, state-transition systems including Markov Decision Processes (MDPs), game-theory based decision models, and path planning methods like search-based algorithms (A* and Dijkstra) and optimization-based techniques.

Data-driven decision and planning methods: This section explores data-driven decision-making and planning methods, covering imitation learning, reinforcement learning, inverse reinforcement learning, and associated challenges. It discusses training systems via expert behavior observation, environment interaction for optimal strategy learning, and inferring reward functions, emphasizing their application and real-world challenges in autonomous driving.

Hybrid decision and planning methods: This section examines hybrid decision-making and planning methods that merge knowledge-driven and data-driven approaches to improve accuracy and efficiency. It discusses the integration of expert knowledge with techniques like imitation and reinforcement learning, highlighting both the benefits of this combination and the technical and practical challenges in implementation.

Experiment platform: This section explores pivotal resources for autonomous driving: datasets and simulation platforms, detailing their sources, composition, applications, and support for decision-making algorithm development. It also evaluates simulation platforms, examining their features and role in testing algorithms and simulating complex traffic environments, crucial for advancing autonomous driving technology.

Challenges and future perspectives: This section addresses current challenges in autonomous driving decision-making and planning, including environmental perception uncertainties, unpredictability of traffic participants, and limitations of data-driven algorithms, and examines industry and academic responses. It also looks to future trends, such as multi-sensor fusion, deep learning, behavior prediction models, scenario simulation, reinforcement learning, synthetic data, continuous learning, and explainable AI.

### 1.3 Significance and contributions

The autonomous driving industry has been progressing at a rapid pace, yet there exists a notable gap in the systematic analysis and synthesis of decision-making and planning methods. Our review aims to bridge this gap by providing a comprehensive and systematic categorization, comparison, and analysis of the current state of the art in autonomous driving. Through this rigorous examination, we have identified a clear trend and a compelling direction for future research: the integration of knowledge-driven and data-driven approaches into hybrid methods.

The lack of systematic analysis in the field has led to fragmented development and a lack of clarity on the most effective strategies for advancing autonomous driving systems. Our review stands as a testament to the need for a structured evaluation of the various methodologies, encompassing rule-based, state transition-based, game-theory based, search-based, sampling-based, and optimization-based methods. By conducting a thorough comparative analysis, we have been able to elucidate the strengths and limitations of each approach and how they complement one another.

Our systematic summary and synthesis have led us to conclude that the future of autonomous driving decision-making and planning lies in hybrid methods. This conclusion is not merely a promotion of a particular approach but is grounded in the recognition that no single methodology can address the multifaceted challenges of autonomous driving. Hybrid methods offer a balanced and comprehensive framework that leverages the strengths of both knowledge-driven and data-driven strategies, thereby enhancing the adaptability, safety, and interpretability of autonomous vehicles.

We advocate for hybrid methods as the future research direction because they hold the potential to: Improve Adaptability: By incorporating data-driven learning, hybrid methods can adapt to dynamic and complex traffic scenarios that exceed the capabilities of traditional rule-based systems.

Enhance Safety and Reliability: Knowledge-driven components provide a safety net, ensuring that decisions comply with predefined rules and ethical standards, which is critical for public trust and regulatory compliance.

Ensure Interpretability: The combination of data-driven flexibility with knowledge-driven structure allows for greater transparency in decision-making processes, which is essential for debugging, optimization, and building user trust.

In conclusion, our systematic analysis and synthesis of autonomous driving decision-making and planning methods provide a clear premise and direction for the field. We believe that the hybrid approach, informed by our comprehensive review, is not only a promising direction but also a necessary evolution in the development of autonomous driving systems. Our review serves as a roadmap for researchers and practitioners, guiding the industry toward a future where autonomous vehicles can operate with enhanced safety, efficiency, and reliability. The contributions of this paper can be summarized as follows:

This paper provides a comprehensive overview of automated decision-making and planning methods, and innovatively classifies these methods into three categories: knowledge-driven methods, data-driven methods, and hybrid methods. Regarding knowledge-driven methods, we highlight the efficiency and accuracy of expert systems in handling specific decision problems through rule system design and state management, and we explore the role of game theory in strategy formation. Additionally, we also discuss how search and optimization algorithms plan paths. For data-driven methods, we analyze decision-making and planning methods based on reinforcement learning, imitation learning, and inverse reinforcement learning. We explore the theoretical foundations, current applications, and challenges of these algorithms. This comprehensive analysis highlights the strengths and limitations of various methods and provides direction for future research and technological improvements. Moreover, we delve into hybrid methods, emphasizing their potential to integrate the advantages of data-driven and knowledge-driven approaches in autonomous driving decision systems.

Furthermore, we detail various virtual simulation platforms and physical experimental facilities necessary for testing and validating algorithms, emphasizing their crucial role in transitioning algorithms from theory to real-world applications.

Finally, we discuss industry challenges and prospects, clarifying future research directions and the integration potential of emerging technologies in the field of automated decision-making and planning.

Overall, this paper enriches academic research in automated decision-making and planning and guides practitioners in the field, contributing positively to technological advancements in this domain.

## 2 Knowledge-driven decision and planning methods

**Figure 1:**
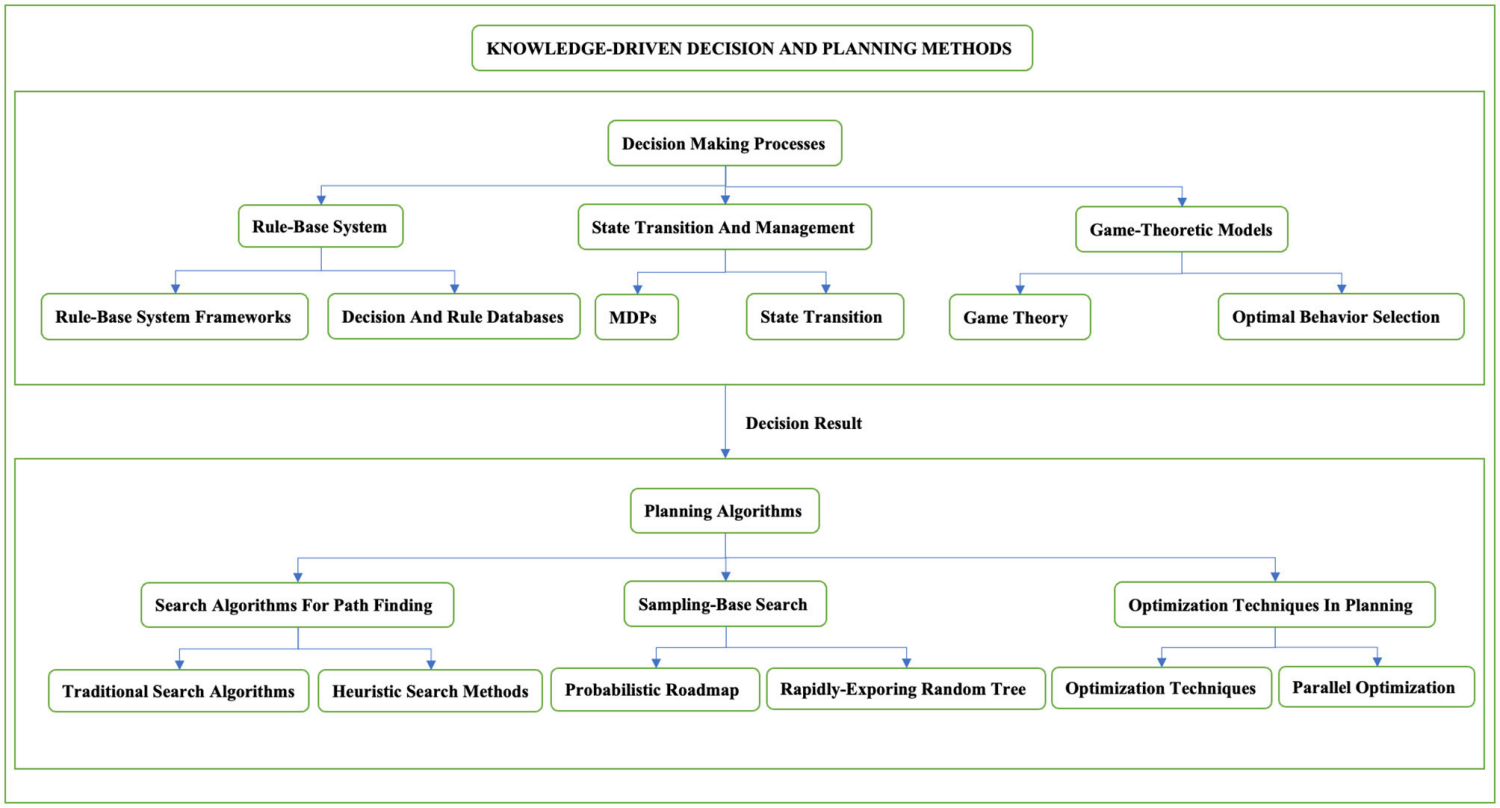
Classification structure of knowledge driven methods. This figure carefully classifies the knowledge-driven decision-making planning methods and introduces the decision-making planning methods in stages. It is mainly divided into two stages, including the decision-making process and the planning process. The second section will discuss each method in the two-stage process in detail.

The knowledge-driven method typically separates the decision-making phase from the path planning phase to enhance modularity, manageability, and efficiency. This distinction allows the decision module to focus on high-level strategies, such as overtaking or following other vehicles, while the path planning module translates these strategies into specific driving paths. The overall classification structure of knowledge driven methods is shown in Figure 1.

The advantage of the knowledge-driven planning method is that it ensures the vehicle remains in a safe state within the predefined range of rules. However, this approach also has several drawbacks, such as overly conservative decision-making and increased time complexity due to the accumulation of rules. While the introduction of trajectory post-processing helps ensure the smoothness and safety of the vehicle's trajectory, the issue of planning delays persists. In the following sections, we will discuss the knowledge-driven planning method in detail, including rule-based and learning-based approaches, examining its benefits, limitations, and potential improvements.

### 2.1 Decision making processes

**Figure 2:**
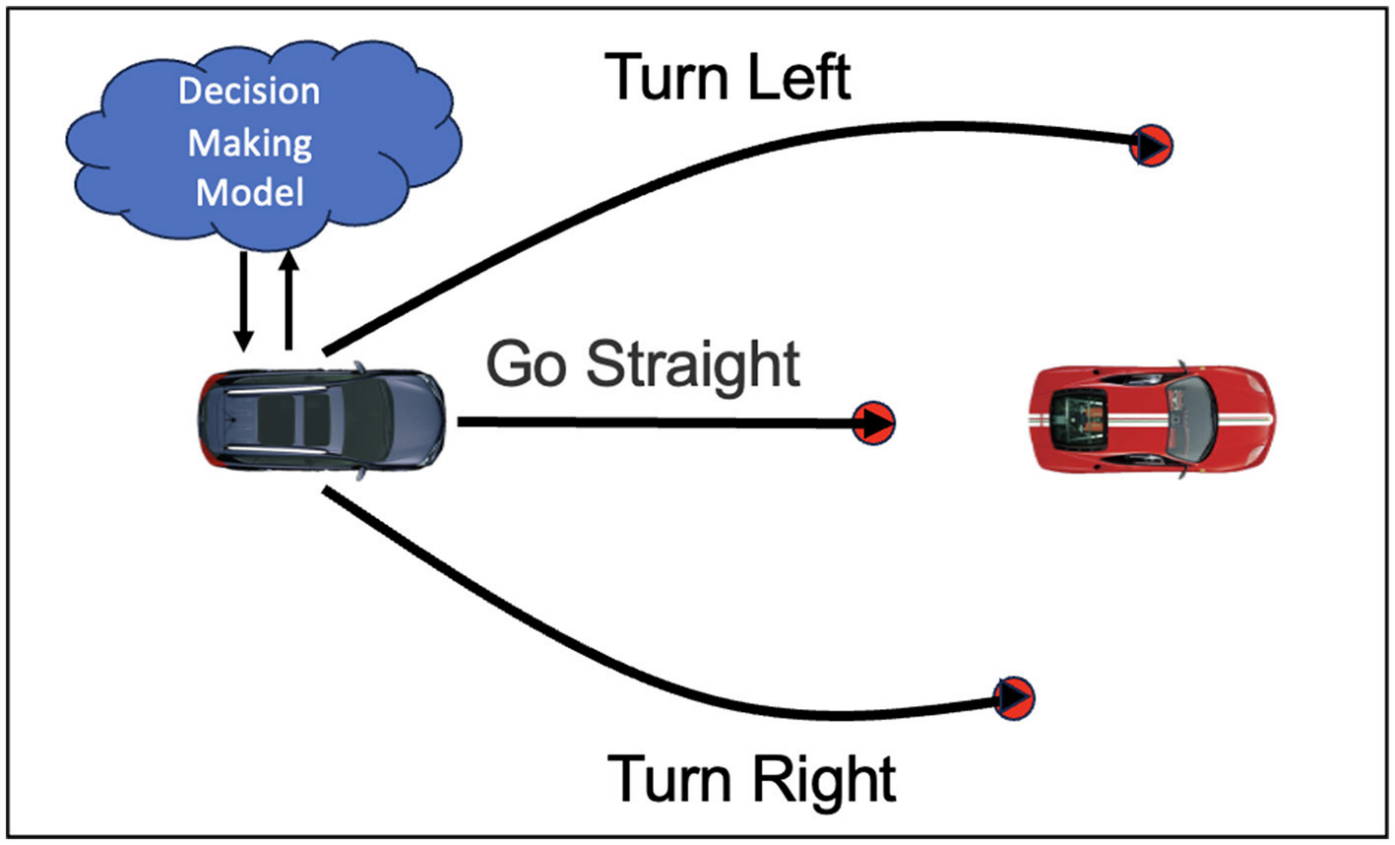
Schematic diagram of knowledge driven decision-making. The figure describes the possible decisions that the vehicle may make when encountering an obstacle vehicle, including going straight, turning left, and turning right. The vehicle plans the optimal trajectory based on the decision-planning model.

The decision module provides initial coarse-grained decision results for the knowledge driven decision and planning model, as shown in Figure 2. Decision-making methods can be categorized into rule based, state-transition based, and game-theory based approaches. Rule based methods use predefined traffic rules and driving strategies, making decisions with conditional logic and reasoning. State-transition methods, such as Markov Decision Processes (MDP) and Partially Observable Markov Decision Processes (POMDP), represent the driving environment and vehicle states as a state space, optimizing state transitions for optimal decisions. Game-theory based approaches treat autonomous driving as a multi-agent system, using game theory to analyze and predict other traffic participants' behaviors to form cooperative or competitive driving strategies. The following sections provide a detailed overview of these decision-making methods.

#### 2.1.1 Rule based decision systems

Rule based systems make decisions with predefined rules and logic. These systems rely on expert knowledge and experience to build decision logic and rules. Zhao et al. (2021) propose a rule based system using if-then rules to process perception information from the traffic environment, generating corresponding driving behaviors. The decision logic of this system is to select the optimal driving strategy based on the surrounding traffic conditions and potential risk assessment. Additionally, the design of the rule database is a crucial component of rule based systems. Pellkofer and Dickmanns (2002) propose a behavior decision module, which execute task plans generated by task planning experts rule database. Hillenbrand et al. (2006) introduce a multi-level collision mitigation system that decides whether to intervene by evaluating the remaining reaction time (TTR). The system employs a time-based decision-making approach, and provides a flexible trade-off between potential benefits and risks while maintaining product liability protection and driver acceptance. Dam et al. (2022) propose an advanced predictive mechanism that comprehensively analyzes the current state of traffic flow and vehicle behavior patterns. This system can anticipate potential upcoming situations by utilizing probabilistic models and machine learning techniques.

In highly complex traffic environments, maintaining system reliability and effectiveness is a critical issue. Zhang T. et al. (2023) address this problem by proposing several solutions, including enhanced perception capabilities, real-time traffic data analysis, adaptive rule adjustment, multimodal decision fusion, safety strategies, redundancy design, and human-machine interaction optimization. Koo et al. (2015) discuss how to enhance the transparency of rule based systems, making their decision-making processes easier to understand and verify. Noh and An (2017) define a decision-making framework for highway environments, capable of reliably and robustly assessing collision probabilities under current traffic conditions and automatically determining appropriate driving strategies. This framework consists of two main components: situation assessment and strategy decision-making. The situation assessment component uses multiple complementary “threat metrics” and Bayesian networks to calculate “threat levels” at both vehicle and lane levels, assessing collision probabilities in specific highway traffic conditions. The strategy decision-making component automatically determines suitable driving strategies for given highway scenarios, aiming for collision-free, goal-oriented behavior.

Trajectory prediction and multi-angle trajectory quality evaluation must be introduced into the rule-based decision-making system. This will allow the drivable trajectory to be planned at future moments more quickly and accurately while ensuring driving safety. The most important thing is to ensure the absolute safety of all agents in a complex environment.

#### 2.1.2 State transition and management models

State transition and management models describe how a vehicle moves between different states. State management involves making decisions based on current state and environmental information. The application of Markov Decision Processes (MDPs) is particularly significant, providing an effective mathematical framework for state management. Galesloot et al. (2024) introduce a novel online planning algorithm to address challenges in multi-agent partially observable Markov decision processes (MPOMDPs). They integrate weighted particle filtering into sample-based online planners, and leverage the locality of agent interactions to develop new online planning algorithms operating on a Sparse Particle Filter Tree. Sheng et al. (2023) introduce a safe online POMDP planning approach that computes shields to restrict unsafe actions violating reach-avoid specifications. These shields are integrated into the POMDP algorithm, presenting four different methods for shield computation and integration, including a decomposed variant aimed at enhancing scalability. Furthermore, Barenboim and Indelman (2024) propose an online POMDP planning method that provides deterministic guarantees by simplifying the relationship between the actual solution and the theoretical optimum. They derived tight deterministic upper and lower bounds for selecting observation subsets at each posterior node of the tree. The method simultaneously constrains subsets of state and observation spaces to support comprehensive belief updates. Ulfsjöö and Axehill (2022) combine POMDP and scenario model predictive control (SCMPC) in a two-step planning method to address uncertainty in highway planning for autonomous vehicles. Huang Z. et al. (2024) propose an online learning-based behavior prediction model and an efficient planner for autonomous driving, utilizing a transformer-based model integrated with recurrent neural memory to dynamically update latent belief states and infer the intentions of other traffic participants. They also employed an option-based Monte Carlo Tree Search (MCTS) planner to reduce computational complexity by searching action sequences. Schörner et al. (2019) develop a hierarchical framework for autonomous vehicles in multi-interaction environments. This framework addresses decision-making under occluded conditions by computing the vehicle's observation range. Additionally, it considers current and predicted environments to foresee potential hidden traffic participants. Lev-Yehudi et al. (2024) introduce a novel POMDP planning approach for target object search in partially unknown environments. Liu et al. (2015) present a trajectory planning method using POMDP to handle scenarios with hidden road users. Chen and Kurniawati (2024) propose a context-aware decision-making algorithm for urban autonomous driving, modeling the decision problem as a POMDP and solving it online.

The utilization of Markov Decision Processes (MDP) and their various extensions in the field of autonomous driving has greatly enhanced the capacity for effective decision-making and strategic planning, particularly in environments that are partially observable or involve multiple interacting agents. In real-world driving scenarios, vehicles often encounter situations where not all variables or conditions are fully visible or predictable, such as obstacles obscured from sensors or dynamic traffic patterns. MDPs provide a structured framework to address these uncertainties by allowing autonomous systems to evaluate potential actions based on probabilistic models of outcomes, thereby optimizing decision-making under uncertainty. Furthermore, in multi-agent environments where interaction with other vehicles, pedestrians, and traffic systems is required, extensions of MDPs, such as Multi-agent MDPs (MMDPs), facilitate coordinated strategies that ensure safe and efficient navigation. These advanced models enable autonomous vehicles to anticipate and respond to the actions of other agents, leading to more reliable and intelligent driving solutions. Overall, the applications of MDP and its variants in autonomous driving enable effective decision-making and planning in partially observable and multi-agent environments.

#### 2.1.3 Game-theoretic models

Game theory plays a critical role in decision-making by analyzing the strategies and potential actions of different participants, enabling autonomous driving systems to optimize their behavior in competitive environments. Fisac et al. (2019) propose a hierarchical dynamic game theory planning algorithm, effectively handling the complex interactions between autonomous vehicles and human drivers by decomposing dynamic games into long-term strategic games and short-term tactical games. Sankar and Han (2020) employ adaptive robust game theory decision strategies within a hierarchical game theory framework to manage vehicle interactions on highways. This strategy allows autonomous vehicles to adjust their behavior based on other drivers' actions, reducing collision rates and increasing lane-changing success. Li et al. (2020), Cheng et al. (2019), and Li et al. (2018) utilize non-cooperative game theory methods to address traffic decision-making at unsignalized intersections. In these methods, each vehicle is viewed as an independent decision-maker to minimize its travel time or enhance its safety without necessarily cooperating with other vehicles. Martin et al. (2023), Tian et al. (2022), and Fang et al. (2024) explore cooperative game theory to enhance the efficiency and safety of interactions among autonomous vehicles. In cooperative game theory, multiple participants form coalitions and share information or resources to achieve common goals, such as reducing overall travel time or increasing overall system safety. The cooperative driving framework allows vehicles to share their location and speed information and predict the intentions and behaviors of others, leading to more coordinated and safer decisions in complex road environments. For autonomous vehicle control at roundabouts, Tian et al. (2018) demonstrate the effectiveness of adaptive game theory decision algorithms by online estimating the opponent driver types and adjusting strategies accordingly, thus managing multi-vehicle interactions in complex traffic environments.

### 2.2 Planning algorithms

**Figure 3:**
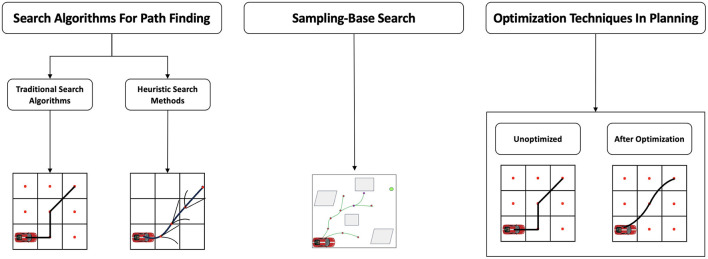
The knowledge-driven algorithm framework is mainly divided into three parts: search-based methods, sampling-based methods, and optimization-based methods. From the figure, the hybrid search effect is more prominent in the search-based methods, and the trajectory planned by the optimization-based method is smoother.

Path planning methods are typically divided into two stages. The first stage includes search-based methods, such as A* and Dijkstra, which systematically search all possible paths to find the optimal solution for well-defined and relative problems. Additionally, it also includes sampling-based methods, such as Rapidly-Exploring Random Trees (RRT) and Probabilistic Roadmaps (PRM), which generate candidate paths through random sampling and are suitable for high-dimensional and complex planning spaces. The second stage primarily employs optimization techniques by designing objective functions and constraints to model the path optimization goals. Ultimately, this process results in a planned path that meets requirements for smoothness, safety, and efficiency. Figure 3 depicts the architectural forms of different planning algorithms.

#### 2.2.1 Search algorithms for path finding

Traditional search algorithms such as A* and Dijkstra systematically explore the path space to find the optimal route from a start to an end point. However, the environments for path planning have become increasingly complex. Diverse heuristic and meta-heuristic search methods have emerged to enhance computational efficiency and path quality. Ferguson and Stentz (2006) explore the field D* algorithm, which calculates path cost estimates during linear interpolation to generate paths with continuous headings. Daniel et al. (2010) find shorter paths on grids without restricting path direction. Wang J. et al. (2019) combine A* with neural networks to incorporate rich contextual information and learn user movement patterns. Meanwhile, Melab et al. (2013) demonstrate the efficiency and practicality of the ParadisEO-MO-GPU framework, a GPU-based parallel local search meta-heuristic algorithm, which implements parallel iterative search models on graphical processing units. Stochastic search algorithms and their variants are also applied in autonomous vehicle path planning to handle dynamic obstacles and complex scenarios. Kuffner and LaValle (2000) solve single-query path planning problems by constructing trees from both the start and end points. Yu et al. (2024a) improve planning efficiency in dynamic environments, which combines dual-tree search with efficient collision detection mechanisms. Huang and Lee (2024) achieve asymptotically optimal path planning in narrow corridors by adaptive information sampling and tree growth strategies.

#### 2.2.2 Sample algorithms for path finding

Sampling-based methods address path planning problems in complex and dynamic environments by generating candidate paths with random sampling in the planning space. Rapidly-exploring Random Trees (RRT) and their variants are representative of these methods. Specifically, Wang Z. et al. (2024) demonstrate that by introducing guided paths and dynamically adjusting weights, which effectively reduces planning time and path curvature. Similarly, Dong et al. (2020) propose a knowledge-biased sampling-based path planning method for Automatic Parking (AP). This approach improves the algorithm's integrity and feasibility by introducing reverse RRT tree growth, using Reeds-Shepp curves to directly connect tree branches, and employing standardized parking space/vehicle knowledge-biased RRT seeds. Chen et al. (2022) propose a strategy combining RRT and Dijkstra algorithms to adapt to semi-structured roads. The method first narrows the planning area using an RRT-based guideline planner, then translates the path planning problem into a discrete multi-source cost optimization problem. The final output path is obtained by applying an optimizer to a discrete cost evaluation function designed to consider obstacles, lanes, vehicle kinematics, and collision avoidance performance. Huang H. et al. (2024) adopt the least action principle to general optimal trajectory planning for autonomous vehicles, offering a method to simulate driver behavior for safer and more efficient trajectory planning. Beyond RRT algorithms, the Probabilistic Roadmap (PRM) algorithm and its improved versions have shown superiority in narrow path planning. Huang Y. et al. (2024) enhance the PRM method by combining uniform sampling with Gaussian sampling, increasing the success rate and efficiency of path planning in narrow passages. Zhang Z. et al. (2023) transform a grid map into a formal context of concepts, mapping the relative positional relationships between rectangular areas. They then convert these relationships into partial order relationships within a rectangular region graph based on concept lattices.

#### 2.2.3 Optimization techniques in planning

Optimization techniques in planning are broadly applied and critical, By appropriately selecting and applying suitable optimization methods, the performance and efficiency of planning systems can be significantly improved. Xu et al. (2012) introduce a real-time motion planner that achieves efficient path planning through trajectory optimization. Similarly, Zhang et al. (2020) decompose the path planning process into two stages: generating smooth driving guidance lines and then optimizing the path within the Frenet frame. Additionally, Werling et al. (2010) combine long-term goals (such as speed maintenance, merging, following, stopping) with reactive collision avoidance. The method demonstrates its capability in typical highway scenarios, generating trajectories that adapt to traffic flow and validating. Zhang Y. et al. (2023) and Gulati et al. (2013) employ nonlinear constrained optimization methods to compute trajectories that comply with kinematic constraints. The method focuses on dynamic factors such as continuous acceleration, obstacle avoidance, and boundary conditions to achieve human-acceptable comfortable motion.

In practical applications, the efficiency and feasibility of implementing optimization algorithms are critical. Stellato et al. (2020) propose the OSQP solver, which effectively addresses convex quadratic programming problems using the Alternating Direction Method of Multipliers (ADMM), making it suitable for real-time applications. High-performance nonlinear optimization has been realized through domain-specific languages (DSL), where GPU acceleration is used to enhance solving efficiency (Yu et al., 2024b). Furthermore, Huang X. et al. (2023) adopt the Levenberg-Marquardt optimization algorithm for nonlinear systems to the control of continuous stirred-tank reactors, showing faster convergence rates and stronger disturbance resistance.

In summary, optimization techniques play a pivotal role in enhancing the efficiency and effectiveness of planning systems, particularly in the context of autonomous driving and real-time applications. By strategically selecting and applying appropriate optimization methods, such as trajectory optimization and nonlinear constrained optimization, these systems can achieve significant improvements in path planning and dynamic response to environmental factors. The integration of advanced solvers like the OSQP for convex quadratic programming and the utilization of domain-specific languages for leveraging GPU acceleration further highlight the importance of optimization in achieving high-performance planning. These approaches demonstrate the capability to address complex scenarios, maintain compliance with kinematic constraints, and ensure comfort and safety in motion, thereby validating their critical role in theoretical and practical applications across various domains.

## 3 Data-driven decision and planning methods

### 3.1 Imitation learning algorithms

Imitation Learning (IL) has become a pivotal methodology in the advancement of Autonomous Vehicles (AVs), leveraging expert demonstrations to navigate around the complexities and hazards intrinsic. Imitation Learning for autonomous vehicles is categorized into three main approaches: Behavioral Cloning (BC), Inverse Reinforcement Learning (IRL), and Generative Adversarial Imitation Learning (GAIL).

Behavioral Cloning (BC) is a straightforward approach that directly mimics human driving behavior. The approach offers several advantages, including simplicity, ease of training, and effective performance when there is a substantial amount of high-quality human driving data available. However, BC encounters difficulties when confronted with unfamiliar road scenarios, is susceptible to noise in the training data, and does not consider the long-term implications of decisions. BC is typically employed in relatively simple and structured environments, such as highways or known routes. Inverse reinforcement learning (IRL) offers the advantage of understanding the underlying intent of human drivers by inferring a reward function, thereby capturing complex driving strategies. This makes it an advantageous approach in diverse and complex scenarios. It demonstrates effective adaptation to novel environments; however, it is associated with considerable computational complexity, necessitating substantial resources and well-designed features and models. IRL is frequently utilized in scenarios that necessitate the comprehension of intricate decision-making processes, such as urban driving. Generative Adversarial Imitation Learning (GAIL) integrates the strengths of generative adversarial networks to enhance model robustness and generalization through adversarial training. GAIL can imitate complex behaviors without explicit reward functions, thereby providing better adaptability to unknown situations. However, its training process can be unstable, involves complex tuning, and demands high-quality and diverse training data. GAIL is suitable for uncertain driving environments that require high robustness and adaptability, such as dynamic urban traffic. An overview of these approaches will be presented in this section.

#### 3.1.1 Imitation learning problem formulation

Imitation Learning approach exploits the vast repository of human driving data to train policies that emulate expert behavior. The fundamental problem definition for IL in the context of AVs revolves around deriving a policy π^*^ that closely matches the expert's policy π^*E*^ by minimizing the discrepancy between their state-action distributions across a dataset **D** of demonstrated trajectories. Each trajectory *t* within **D** consists of sequential state-action pairs (*s*_*it*_,*a*_*it*_), where *a*_*it*_ is the action executed by the expert in state *s*_*it*_ according to π^*E*^. The optimization framework for achieving this can be mathematically formalized as:


(1) $$\begin{array}{l} \pi^{*}=arg\;\underset{\pi }{\min}L\left(\pi^{E},\pi \right) \end{array}$$

where L is a divergence measure that quantifies the dissimilarity between the expert's policy and the learned policy.

BC simplifies the IL challenge by transforming it into a supervised learning problem, with the aim of learning a policy. π_θ_ that minimizes the loss function L over the dataset **D** of state-action tuples, thereby replicating the expert's behavior:


(2) $$\begin{array}{l} \pi_{\theta }^{*}=arg\;\underset{\theta }{\min}E_{\left(s,{\text{a}}^{E}\right)\sim P^{E}\left(s|\pi^{E}\right)}L\left(a^{E}-\pi_{\theta }\left(s\right)\right) \end{array}$$

Where the *P*^*E*^(*s* ∣ π^*E*^) is the state distribution of the expert policy, and the L can be a loss function that measures the imitation quality of the expert's actions. Commonly, L can be the L1 loss (mean absolute error) or the L2 loss (mean squared error). Taking L2 loss as an example, the loss function can be:


(3) $$\begin{array}{l} Loss=\frac{1}{T}\sum_{j=1}^{T}{\left\|a-a^{E}\right\|}_{2} \end{array}$$

The problem of IRL in autonomous driving revolves around inferring a reward function r^*^ from expert demonstrations. Given a set of state-action pairs sampled under the expert policy π^*^, IRL attempts to learn a reward function r^*^ such that:


(4) $$\begin{array}{l} \pi^{*}=arg\;\max E_{\pi }\left[r^{*}\left(s,a\right)\right] \end{array}$$

Generative Adversarial Imitation Learning (GAIL) is a framework that extracts expert-driving policies without explicitly defining reward functions or employing laborious reinforcement learning cycles. It synergistically combines imitation learning with Generative Adversarial Networks (GANs), creating a duel between a generator and a discriminator. The generator, parameterized by θ, emulates expert maneuvers by matching the distribution of state-action pairs observed in demonstrations. Meanwhile, the discriminator represented by *D*_ω_ within the interval (0, 1) serves as a stand-in reward evaluator, quantifying the likeness between the generated and actual expert behaviors. The GAIL lies in a min-max optimization objective, succinctly expressed as:


(5) $$\begin{array}{l} \underset{\pi_{\theta }}{\min}\underset{D_{\omega }}{\max}{\text{E}}_{\pi_{\theta }}\left[\log D_{\omega }\left(s,a\right)\right]+{\text{E}}_{\pi_{E}}\left[\log\left(1-D_{\omega }\left(s,a\right)\right)\right]-\lambda H\left(\pi_{\theta }\right) \end{array}$$

This formula pits the generator against the discriminator, where **E**_π_θ__[log*D*_ω_(*s, a*)] encourages the generator to produce actions indistinguishable, and ensures the discriminator's sharpness in distinguishing real from fake samples. The term **E**_π_*E*__[log(1 − *D*_ω_(*s, a*))] introduces entropy regularization to promote policy exploration. Here, *H*(π_θ_) denotes the entropy of policy π, a measure of randomness in action selection that fosters learning flexibility. Gradients guiding updates for both components are defined as:


(6) $$\begin{array}{l} \nabla_{\theta }J\left(\theta \right)={\text{E}}_{\pi }\left[\nabla_{\theta }\log\pi_{\theta }\left(a|s\right)Q\left(s,a\right)\right]-\lambda \nabla_{\theta }H\left(\pi_{\theta }\right) \end{array}$$

(7) $$\begin{array}{l} \nabla_{\omega }J\left(\omega \right)={\text{E}}_{\pi }\left[\nabla_{\omega }\log D_{\omega }\left(s,a\right)\right]+{\text{E}}_{\pi_{E}}\left[\nabla_{\omega }\log\left(1-D_{\omega }\left(s,a\right)\right)\right] \end{array}$$

#### 3.1.2 Behavior clone methods

**Figure 4:**
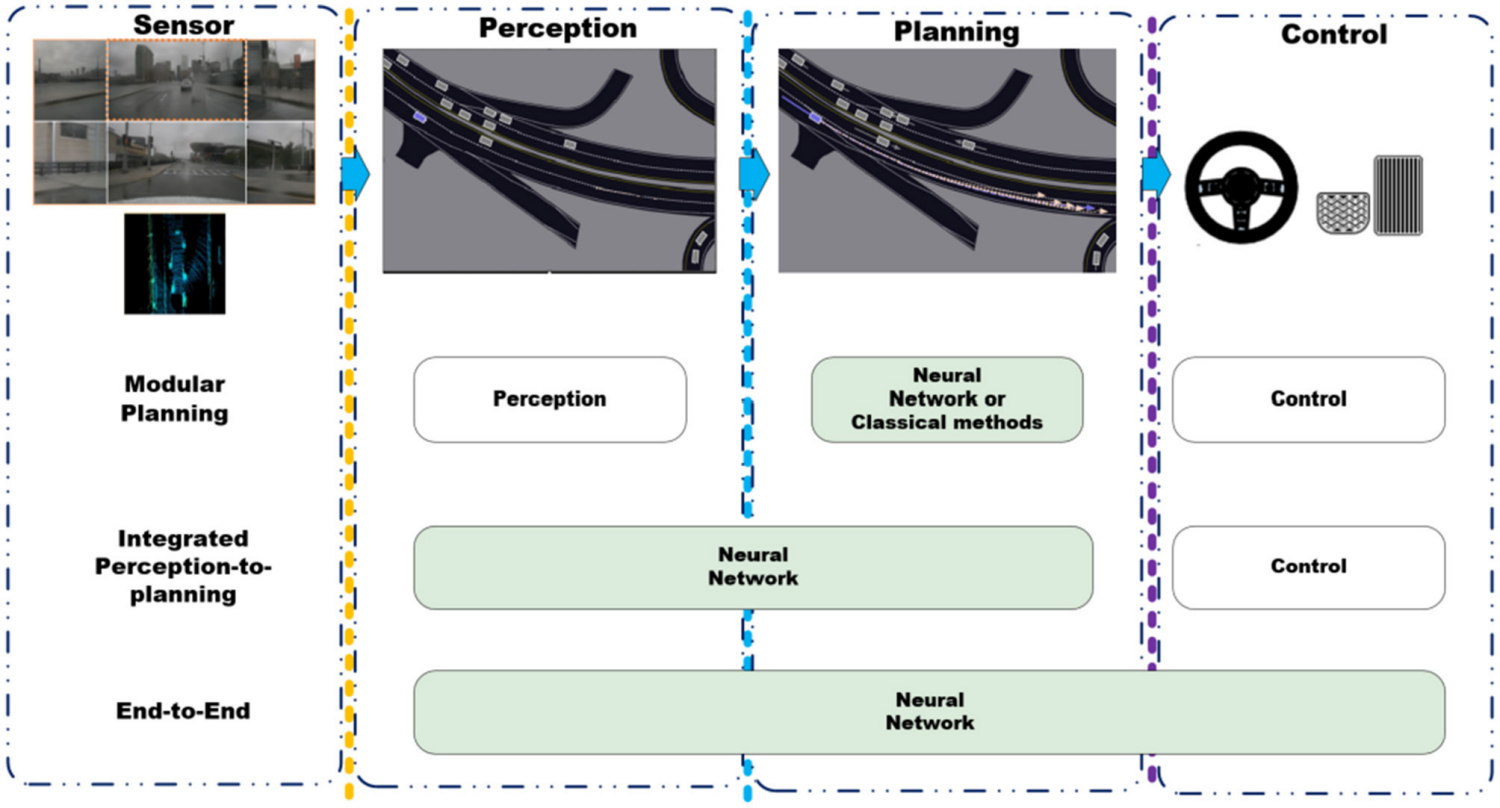
The taxonomy of Behavioral Cloning. Autonomous driving tasks are composed of three key components: perception, planning, and control. Perception generates environmental perception outcomes based on sensor inputs. Planning creates the future trajectory of the vehicle by considering perception results and historical state information. Control implements vehicle control actions according to the planning outcomes. Regarding the taxonomy of Behavioral Cloning methods within the context of planning task, it can be categorized into three types. Modular Planning links the perception, planning, and control modules in a pipeline fashion. Integrated Perception-to-planning integrates the perception and planning tasks into a single module. End-to-End indicates that the entire perception, planning, and control tasks are accomplished by a unified module.

We refine the taxonomy of Behavioral Cloning into two unique categories, as shown in Figure 4: End-to-End and Modular Planning.

##### 3.1.2.1 End-to-End methods

End-to-End methodologies involve models that are optimized to infer precise steering and acceleration commands directly from raw sensor data. ALVINN (Pomerleau, 1988) was the first implementation of end-to-end Imitation Learning for autonomous driving in 1989. There is a front-facing camera installed on ALVINN as visual input, and it uses a 3-layer MLP as the policy function approximator. ALVINN learns in real time using data from the human driver to train for lane-keeping steering commands. The AVLINN is extended with obstacle avoidance by Muller et al. (2005). It presents an obstacle avoidance system for small off-road vehicles equipped with twin forward-facing cameras named DAVE. Using end-to-end learning, the system is trained on raw image data coupled with human driver input in a variety of environments. The work from NVIDIA (Bojarski et al., 2016) has taken the concept of end-to-end imitation learning a step further with its DAVE-2, which uses inputs from three onboard cameras. The dual perspective provided by the offset left and right cameras enables the system to correct for vehicle drift. A further work from NVIDIA (Bojarski et al., 2017) provides an exhaustive analysis of the interpretability of the neural network for autonomous driving proposed by Bojarski et al. (2016). The core of this research is to reveal how the deep neural network decides the driving direction based on the input road images, particularly in the context of an end-to-end learning framework. Another work (Cultrera et al., 2020) proposes an explainable autonomous driving system using imitation learning with visual attention. By integrating an attention mechanism, the system highlights important image sections to enhance decision transparency. Hecker et al. (2018) present an end-to-end driving model utilizing eight surround-view cameras strategically mounted for a 360-degree perspective. The model integrates CNNs for feature extraction and LSTM layers for temporal encoding. Codevilla et al. (2018) propose Conditional Imitation Learning (CIL), a framework that enriches the learning process by incorporating explicit intent information alongside visual observations. The CIL model is designed to receive not only visual data from a three-camera configuration–inspired by the DAVE-2 system (Bojarski et al., 2016), but also a high-level command indicating the intended action (e.g., turn left, turn right, go straight). Their network comprises convolutional layers for feature extraction, followed by Long Short-Term Memory (LSTM) units to handle temporal sequences.

The efficacy of the CIL framework is assessed through a dual-pronged evaluation approach. Hawke et al. (2020) advance the application of Conditional Imitation Learning (CIL) by presenting an end-to-end autonomous driving system proficient in executing both steering and speed adjustments amidst intricate urban landscapes. To infuse temporal awareness, an optical flow model is incorporated by Sun D. et al. (2018), enhancing the system's understanding of motion dynamics. Xiao et al. (2020) develop an end-to-end autonomous vehicle framework that integrates both RGB imagery and depth data from onboard LiDAR sensors. Wang Q. et al. (2019) employ the vehicle's current location and intended trajectory to compute a “subgoal angle,” which serves as an input to the neural network. The network takes sequential images, vehicle speed, and the computed subgoal angle as inputs. Its initial seven layers are pre-trained on ImageNet to facilitate feature extraction. Separate feature extractor modules process the input images, speed, and subgoal angle independently. These extracted features are subsequently combined to forecast steering commands and throttle inputs. Haavaldsen et al. (2019) delve into the integration of recurrent layers within end-to-end autonomous vehicle architectures. It involves the training of two distinct models: a standard Convolutional Neural Network (CNN) operating in an end-to-end framework and a CNN with recurrent layers. The training data contains 3 camera images, traffic signals, a high-level command, output steers, and speed control signals. Chi and Mu (2017) introduce a model that incorporates LSTM architecture to enhance its capacity for temporal reasoning. This model treats the steering angle as a dynamically evolving variable, reflecting the continuity inherent in driving actions. The integration of LSTM within a dual-subnetwork framework ensures a comprehensive understanding of the driving environment and historical vehicle dynamics. Kebria et al. (2019) uncover that models with increased depth surpass their less deep counterparts, with a substantial improvement particularly evident when transitioning from 9 to 12 layers. Furthermore, models incorporating a varied assortment of filter sizes emerged as the top performers, highlighting the advantage of filter size diversity. Barnes et al. (2017) integrate video odometry for tracking vehicle movement and employs LiDAR for detecting impediments. By merging visual cues from the camera with spatial data from LiDAR, the system can partition the incoming visuals into three classifications at the pixel level: traversable paths, non-traversable areas, and unclassified zones. Cai et al. (2019) propose a model that integrates camera visuals, high-level navigational commands, and past trajectories of autonomous vehicles. This model is structured with three distinct sub-networks, each dedicated to executing a fundamental maneuver: maintaining a straight course, turning left, or turning right. These sub-networks are concatenated with Long Short-Term Memory (LSTM) and Fully Connected (FC) layers to formulate trajectories. Bansal et al. (2018) transform bird's-eye view environmental inputs into driving commands. To overcome the limitations of standard behavior cloning, the model integrates synthesized data simulating challenging scenarios such as collisions and off-road incidents. By augmenting the loss function to penalize unwanted events and encourage progression, the system learns from both ideal and adverse behaviors, achieving higher robustness. Caltagirone et al. (2017) propose a LiDAR-based driving path generation approach using a fully convolutional neural network (FCN) that integrates LiDAR point clouds, GPS-IMU data, and Google navigation instructions. The system learns to perform perception and path planning directly from real-world driving sequences. This learning-based method bridges low-level scene understanding with behavioral reflexes, enhancing autonomous vehicle technology by producing human-interpretable outputs for vehicle control. Xu et al. (2017) present an end-to-end learning framework for autonomous vehicles with a large, uncalibrated, crowd-sourced video dataset to mitigate the Cascading Error Problem. Their model integrates spatial and temporal cues for continuous steering angle prediction. Model assessment entails contrasting the highest probability predicted action against the actual action for validation. Unlike approaches that directly use a single network to fit autonomous driving tasks, some researchers have introduced more complex multi-stage architectures to enhance network performance. Chen et al. (2020) outline a novel two-stage training method for autonomous driving systems named “Learning by Cheating.” Initially, a “privileged” agent is trained with access to ground-truth environmental data, providing an unrealistic advantage akin to “cheating.” This agent then teaches a “sensorimotor” agent, which operates solely on visual input, mimicking expert behavior without direct access to the privileged information. This strategy breaks new ground by separating perception from decision-making, enabling the vision-based agent to excel without needing explicit environmental cues. Chen and Krähenbühl (2022) present a pioneering system that leverages the experiences of not just the ego-vehicle but also surrounding vehicles for autonomous driving policy training. This innovative approach enriches the diversity of driving scenarios without requiring additional data collection. Wu et al. (2022) propose an integrated solution for autonomous vehicles, merging trajectory planning and control prediction into one system. Shao et al. (2023b) process intricate urban traffic scenarios by incorporating both temporal and global reasoning mechanisms. This system uniquely addresses the challenges of predicting future object movements and managing obscured entities, enhancing safety through superior anticipation of potential hazards in complex situations. Hu et al. (2023b) propose a novel framework that departs from conventional autonomous driving architectures by focusing on planning as the objective. UniAD integrates perception, prediction, and planning tasks into one unified network. The network's unique design revolves around transformer decoder-based modules that facilitate multi-task cooperation and emphasize a planning-first mindset.

##### 3.1.2.2 Modular planning

In contrast to the aforementioned end-to-end architectures that use data-driven methods to model the entire autonomous driving system, Modular Planning focuses on the data-driven aspects, specifically within the decision-making and planning components. Chen et al. (2019) address the challenges associated with conventional model-based decision-making systems. By learning from offline expert driving datasets, the proposed method bypasses the need for manual policy design, thereby offering scalability and adaptability to diverse road conditions, including varying topologies, geometries, and traffic regulations. Sun L. et al. (2018) present planning and control framework, addressing the challenges of real-time, safety, and efficiency. Renz et al. (2022) deviate from conventional pixel-based planning systems that often struggle with efficiency and interpretability in complex environments. PlanT employs an object-centric representation, processing a compact set of scene elements rather than dense grids, thereby enhancing both computational speed and decision transparency. Guo et al. (2023) innovate urban autonomous driving by tackling the covariate shift issue in behavior cloning. It introduces a policy mapping context states directly to ego vehicle trajectories, bypassing combined state-action predictions. Cheng et al. (2023) address the inefficiencies arising from the lack of a standardized benchmark in evaluating imitation-based autonomous driving planners. By leveraging the newly introduced nuPlan dataset and its closed-loop benchmarking framework, they conduct an extensive analysis focusing on two key aspects: crucial features for ego-motion planning and effective data augmentation strategies to mitigate compounding errors. Cheng et al. (2024a) propose a query-based model that integrates lateral and longitudinal self-attentions sequentially, followed by a cross-attention mechanism that aligns the decoded trajectory with the scene context. To mitigate computational intensity, PLUTO employs factorized attention, reducing complexity without sacrificing expressiveness.

#### 3.1.3 Inverse reinforcement learning methods

Inverse Reinforcement Learning (IRL) recognizes reward structures from expert demonstrations that are critical for emulating nuanced human driving behaviors in autonomous vehicles. While Reinforcement Learning (RL) optimizes actions based on known rewards, Inverse Reinforcement Learning (IRL) deduces the underlying reward functions from observed behaviors, providing valuable insights into decision-making processes.

Rosbach et al. (2019) optimize driving styles in an integrated general-purpose planner for autonomous vehicles. The learning process uses human demonstration data, approximating feature expectations within the planner's graph representation to facilitate maximum entropy IRL. Sadigh et al. (2016) adopt IRL to develop strategies for autonomous vehicles that proactively shape the behavior of human drivers. By approximating the human driver as an optimal planner with reward functions learned from demonstrations, the method optimizes robot actions without explicit communication coding. Brown and Niekum (2018) address the challenge of deriving high-confidence performance bounds in scenarios, where the reward function is unknown by employing a Bayesian Inverse Reinforcement Learning (IRL) sampling method. Using demonstration data, the framework employs Markov Chain Monte Carlo techniques to sample reward functions. These samples are used to compute a tight upper bound on the worst-case performance discrepancy between any evaluated policy and the optimal policy induced by the expert's latent reward structure. Palan et al. (2019) employ a novel hybrid framework, which synergistically integrates human demonstrations and preference queries for efficient reward function learning. Lee et al. (2022) adopt an IRL-based spatiotemporal approach for Model Predictive Control (MPC), utilizing a deep neural network structure with goal-conditioning. This network design ingests concatenated bird's eye view images, encompassing occupancy, velocity, acceleration, and lane information, to implicitly learn an interpretable reward function. Cai et al. (2021) adopt IRL to harmoniously blend imitation learning with model-based reinforcement learning techniques. Phan-Minh et al. (2022) employ a unique structured neural network, processing separated features with masked self-attention before integration. Liang et al. (2018) employ a Controllable Imitative Reinforcement Learning (CIRL) methodology. The architecture features a gating mechanism enabling conditional policy execution based on distinct commands, processing raw visual inputs to output continuous control actions like steering angles. Huang et al. (2023c) use IRL for conditional predictive behavior planning in autonomous vehicles. It features a Transformer-based network structure that integrates future ego plans with agent history and vectorised map.

#### 3.1.4 Generative adversarial imitation learning methods

Generative Adversarial Imitation Learning (GAIL) innovatively combines the strengths of imitation and generative adversarial networks (GANs) for autonomous vehicle policy learning. Unlike traditional reinforcement learning, GAIL learns policies directly from expert demonstrations. It employs a two-part structure: a generator acting as a policy and a discriminator acting as a reward function. With GAIL, autonomous systems can learn sophisticated behaviors in an end-to-end manner, improving their adaptability and performance in the field. Li et al. (2017) extend GAIL for unsupervised discovery of latent structures in expert demonstrations. By leveraging visual data, it learns interpretable representations directly from raw pixel inputs. Kuefler and Kochenderfer (2017) extend the InfoGAIL algorithm to address multi-modal imitation learning for sustained behavioral replication. By introducing “burn-in demonstrations,” the method conditions policies at test time, enhancing their ability to mimic expert behavior over extended periods. Kuefler et al. (2017) utilize GAIL to emulate human highway driving patterns. In particular, the performance of this method is significantly better on longer-term predictions (over 3 s), highlighting the superiority under a wide range of assessment metrics. Merel et al. (2017) introduce a novel extension to GAIL, specifically tailored for extracting human-like behaviors from sparse and noisy motion capture data. This method innovates by successfully training policies using partial state features alone. It further demonstrates the feasibility of imitation across dissimilar body structures and dynamics. By integrating a context variable into the GAIL framework to manage multi-behavior policies, the approach fosters seamless transitions between various learned behaviors. Sharma et al. (2018) propose a framework for learning hierarchical policies from unsegmented demonstrations. Directed-info Gail adopts directed information flow in a graphical model to uncover subtasks without the need for action labels. Using an L2 loss, it refines action replication in complex tasks and demonstrates effectiveness in various environments, including grid navigation and continuous control scenarios such as hopper and walker. Fei et al. (2020) introduce a novel approach to the multi-modal GAIL method. It integrates an auxiliary skill selector, enabling the system to adaptively choose behaviors in response to varying contexts. Theoretical convergence guarantees for both the generator and selector ensure optimal policy learning.

### 3.2 Reinforcement learning

RL is primarily classified into three methodologies: value-based, policy-based, and actor-critic methods. Value-based methods, such as Q-learning, concentrate on estimating the value of actions in given states in order to derive optimal policies. The principal benefit of these methods is their simplicity and efficacy in discrete action spaces, rendering them well-suited to environments where the state-action space can be accurately represented and managed. However, they frequently encounter difficulties when confronted with extensive action spaces, a phenomenon known as the curse of dimensionality. Furthermore, they necessitate a considerable amount of exploration to reach optimal policies. These methods are typically employed in scenarios with a finite set of actions, such as grid-world problems or simplified driving tasks. In contrast, policy-based methods directly parameterise and optimize the policy itself, thereby offering advantages in the handling of continuous action spaces and enabling more direct learning of stochastic policies. These methods are particularly effective in environments where the action space is continuous or high-dimensional, such as robotic control or complex maneuvering tasks. However, policy-based methods may be less sample-efficient and may exhibit high variance during training, necessitating the careful tuning of learning rates and exploration strategies. Actor-critic methods integrate the advantages of both value-based and policy-based techniques by employing two distinct structures: the actor, which updates the policy, and the critic, which assesses the action taken by the actor. This combination enables more stable and efficient learning, reducing variance and improving convergence rates. Actor-critic methods are versatile and can be applied in a wide range of settings, from relatively simple tasks to those of a more complex nature, such as autonomous driving in dynamic environments. However, they are prone to being computationally intensive and require careful balancing between the updates to the actor and critic in order to maintain stability.

#### 3.2.1 Reinforcement learning problem formulation

Reinforcement Learning (RL) constitutes a foundational mathematical framework grounded in the principle of trial-and-error learning. Mathematically refined of RL, is formalized as (S, A, P, R, γ), with S and A representing the sets of all possible states and actions, respectively. The transition dynamics function, P(*s*_*t*+1_ ∣ *s*_*t*_, *a*_*t*_): S × S × A → [0, 1], maps state-action pairs to a probability distribution over subsequent states. The instantaneous reward function, R(*s*_*t*_, *a*_*t*_, *s*_*t*+1_): S × A × S → R, furnishes the learning cues. A discount factor γ ∈ [0, 1] governs the present valuation of prospective rewards, with lower values promoting shortsighted decision-making.

For scenarios where some environments are not fully observable, they incorporate an observation space Ω and an observation function O, such that O(*a*_*t*_, *s*_*t*_ + 1, *o*_*t*+1_) = P(*o*_*t*+1_ ∣ *a*_*t*_, *s*_*t*+1_) quantifies the likelihood of perceiving *o*_*t*+1_ following the execution of action at leading to state *s*_*t*+1_.

**Figure 5:**
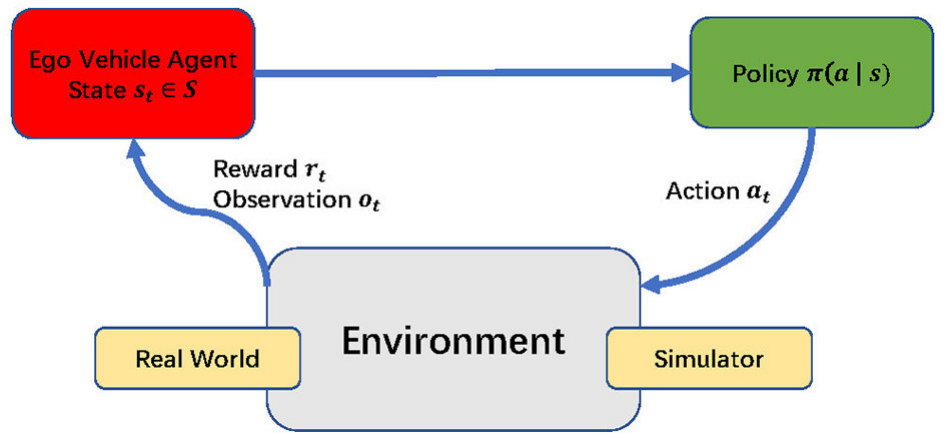
The reinforcement learning processes.

At each discrete time step *t*, the agent conditioned on its present state *s*_*t*_ chooses an action at from the action set A, subsequently earning a numerical reward *r*_*t*+1_ and transitioning to a new state *s*_*t*+1_, as shown in Figure 5. The sequential record {*s*_0_, *a*_0_, *r*_1_, *s*_1_, *a*_1_, *r*_2_, ...} formed is termed a rollout or trajectory. The anticipated accumulation of future rewards, encapsulated by the expected discounted return *G*_*t*_ beyond time step *t*, is mathematically defined as follows:


(8) $$\begin{array}{l} G_{t}≐r_{t+1}+\gamma r_{t+2}+\gamma^{2}r_{t+3}+...=\overset{T}{\underset{K=0}{\sum }}\gamma^{K}r_{t+k+1} \end{array}$$

where T represents a finite value for problems with a finite horizon, and ∞ for those with an infinite horizon. The policy π(*a* ∣ *s*) assigns probabilities to each potential action based on the current state. Meanwhile, the value function under policy π, denoted *v*_π_(*s*), estimates the expected cumulative return when starting from state s and adhering to policy π thereafter.


(9) $$\begin{array}{l} v_{\pi }\left(s\right)≐{\text{E}}_{\pi }\left[G_{t}|s_{t}=s\right] \end{array}$$

Similarly, the action-value function *q*_π_(*s, a*) is defined as:


(10) $$\begin{array}{l} q_{\pi }\left(s,a\right)≐{\text{E}}_{\pi }\left[G_{t}|s_{t}=s,a_{t}=a\right] \end{array}$$

which satisfies the recursive Bellman equation:


(11) $$\begin{array}{l} q_{\pi }\left(s_{t},a_{t}\right)≐{\text{E}}_{s_{t+1}}\left[r_{t+1}+\gamma q_{\pi }\left(s_{t+1},\pi \left(s_{t+1}\right)\right)\right] \end{array}$$

The objective of RL is to identify the optimal policy that maximizes the expected return $\pi^{*}=argmax_{\pi }{\text{E}}_{\pi }\left[G_{t}|s_{t}=s\right]$.

RL is primarily classified into three methodologies: value-based, policy-based, and actor-critic methods.

#### 3.2.2 Value-based RL methods

Value-based RL focuses on assessing the value of states or state-action pairs to guide decision-making. By iteratively updating a value function that predicts future rewards, these methods prioritize actions associated with the highest expected return. Alizadeh et al. (2019) adopt DQN (Deep Q-Network) to address a discrete action space where decisions such as lane changes are made in autonomous driving simulations. Reward mechanisms are designed to incentivise safe behavior, penalize collisions or aggressive maneuvers and encourage goal-directed navigation. Deshpande and Spalanzani (2019) address the challenge of autonomous in the presence of pedestrians. The action space encompasses velocity adjustments and steering decisions, while the state space is depicted through a grid-based representation encoding vehicle and pedestrian positions. Reward mechanisms are in place to encourage safe distances from pedestrians and adherence to traffic rules. Tram et al. (2019) integrate DQN with Model Predictive Control (MPC) to address intersection navigation. The action space comprises six distinct actions, including proceeding through the intersection and yielding. The state space encompasses the dynamic vehicle conditions and partial observability of surrounding traffic. A reward mechanism is devised to ensure that driving practices are both safe and effective. Li and Czarnecki (2018) employ a multi-objective Deep Q-Network variant to address the challenge of autonomous driving. The action space encompasses a wide range of maneuvers, including lane changes and adhering to traffic rules at intersections. The model employs a neural network architecture featuring shared and specialized layers, with inputs including vehicle states and environmental factors. Auxiliary factored Q-functions are integrated to enhance learning efficiency, leveraging structured representations of the environment. Ronecker and Zhu (2019) address the complexity of decision-making in autonomous driving on highways. The action space encompasses the selection of target points for trajectory planning. The reward functions have been designed to align with the specific requirements of highway scenarios. The method provides incentives for safe and efficient driving behaviors, including maintaining speed, executing smooth lane changes, and avoiding collisions. Yuan et al. (2019) employ a Multi-Reward Architecture based DQN approach. The reward functions have been structured in such a way as to incentivise the maintenance of a certain speed, overtakes and safe lane changes. A distinctive network design incorporates a shared low-level with three distinct high-level branches, each dedicated to a specific reward aspect. The training process encompasses a diverse range of scenarios that have been simulated for autonomous highway driving. Liu et al. (2019) employ the DDQN (Double Deep Q-Network) for reinforcement learning, navigating within a discrete action space comprised of semantic actions. Rewards are tailored for safe and efficient maneuvers. Min et al. (2019) employ the DDQN (Distributional Deep Q-Network), which operates in a discrete action space characterized by highway driving scenarios. The rewards are designed for safe and efficient navigation. The network architecture integrates convolutional layers for processing camera images and additional layers for LiDAR data fusion. A distinctive dual Q-function mechanism serves to prevent value overestimation, thereby enhancing the stability of the training process. The model undergoes rigorous learning phases, validated through simulations in a Unity-based highway driving environment. Shi et al. (2019) employ an HDQN (Hierarchical DQN) approach, which addresses the complexity of autonomous lane change tasks in dynamic environments. The rewards are shaped by safety and feasibility metrics. The method employs a dual-layer network structure, with fully connected layers used for decision-making. Through iterative learning and rigorous training, the model demonstrates its proficiency via simulations, exhibiting convergent loss curves and accumulating rewards that signify effective decision-making and planning.

#### 3.2.3 Policy-based RL methods

Policy-based RL directly optimizes a policy function, mapping states directly to action probabilities. This approach adjusts the policy parameters based on the policy gradient. Policy-based methods naturally handle continuous action spaces and can induce more complex behaviors, yet they may suffer from higher variance during optimization. Osiński et al. (2020) employ PPO (Proximal Policy Optimization) to optimize steering commands in a continuous action. The network is trained predominantly on synthetic data, which is more cost-effective than training on real data. The results of real-world validation demonstrated remarkable success in sim-to-real transfer, evidenced by the performance of the system in nine diverse driving scenarios, totalling 2.5 km. Belletti et al. (2017) encompass a variety of policy update methods, with PPO exhibiting a faster convergence to optimal policies. Tang (2019) employ PPO for learning multi-agent negotiations in complex environments. The study addresses a continuous action space, where agents perform tasks such as acceleration, steering, and signaling in a state space that encompasses dynamic traffic scenarios. The rewards are designed to encourage safe and efficient navigation, with penalties for collisions and incentives for adherence to traffic rules. Jang et al. (2019) train autonomous vehicles (AVs) to navigate traffic in a continuous action space with the TRPO (Trust Region Policy Optimization) algorithm. The objective of the reward signals was to minimize traffic delays and to promote smooth merging behaviors. The neural networks process inputs and apply nonlinear transformations for decision-making. Chen et al. (2018) adopt a deep HRL (hierarchical reinforcement learning) approach to address the challenge of autonomous driving tasks with distinct behaviors, such as passing or stopping at traffic lights. The reward system is dynamically adjusted based on the vehicle's action and the factors of vehicle velocity, distance to crossing line, and time till signal change. The hierarchical structure comprises distinct modules for decision-making levels, with inputs including vehicle dynamics and the generation of acceleration commands through a policy network.

#### 3.2.4 Actor-critic methods

Actor-critic algorithms integrate the strengths of both methods: an “actor” generates actions based on learned policies, while a “critic” evaluates these actions through a value function. Actor-critic methods often achieve greater stability and learning efficiency, particularly in complex tasks. Next, we will explore in detail the application of these three methods in decision-making and planning for autonomous driving. Wu et al. (2018) address the challenge of dynamically assigning varying speed limits across lanes, which requires consideration of a complex state space that encompasses multiple traffic factors. The method employs Deep Deterministic Policy Gradient (DDPG), a variant of reinforcement learning, which operates in continuous action spaces. The reward signals encompass efficiency metrics, safety indicators, and environmental impact through emissions. The DDPG model employs a sophisticated actor-critic architecture that utilizes inputs reflecting real-time traffic conditions to learn optimal speed limit adjustments. The training process optimizes both the actor and critic via temporal difference errors and deterministic policy gradients. Gao and Chang (2021) use the Soft Actor-Critic(SAC) algorithm to model autonomous driving system. A ResNet-34 architecture serves as the backbone for both actor and critic networks, processing raw image states coupled with vehicle speed as inputs. Imitation learning pre-training is adopted to improve model initialization before reinforcement learning fine-tuning for optimal performance. Chu et al. (2019) employ Advantage Actor-Critic (A2C) to address the challenge of large, discrete action space inherent in traffic signal control. Utilizing Long Short-Term Memory (LSTM) networks, the model processes complex spatio-temporal traffic flows, maintaining historical context without overwhelming the state representation. The model proposed by Lin et al. (2018) is also an A2C method operating in a discrete action space, where decisions involve switching or maintaining traffic light phases. The state space includes a 2-D tensor reflecting the number of stopped vehicles and average speeds across a 3 × 3 intersection grid. Mousavi et al. (2017) use deep policy gradient algorithms to control traffic signal operations. The model operates in a discrete action space, dictating traffic signal phases and navigating high-dimensional state spaces derived from complex urban traffic dynamics.

## 4 Hybrid decision and planning methods

Hybrid methods exhibit unique advantages in the decision-making and planning of autonomous driving, yet they also encounter numerous challenges. Their merit lies in the successful integration of the learning capabilities of data-driven approaches and the characteristics of knowledge-driven methods. On one hand, the knowledge-driven component furnishes a rule framework that ensures the legality, consistency, and interpretability of decisions, enabling autonomous driving behaviors to adhere to traffic regulations and common sense. On the other hand, the data-driven part, by virtue of learning and mining from vast amounts of data, accurately identifies and adapts to complex and variable scenarios. Algorithms such as EPSILON and MARC, through the collaboration of deep learning models and tree models, significantly enhance the decision-making level, accomplishing flexible, safe, and highly interactive decision planning and strengthening the system's capacity to cope with diverse road conditions and the variety of traffic participants.

Nevertheless, the limitations of hybrid methods cannot be overlooked. In terms of technology integration, due to the disparate underlying principles, data processing logics, and model architectures between data-driven and knowledge-driven technologies, compatibility issues readily emerge during integration, leading to a substantial increase in system complexity. For instance, when combining deep learning models with rule decision trees, differences in data formats, learning modes, and reasoning logics can easily trigger system malfunctions or performance degradation. Regarding stability and reliability, potential conflicts between data and rules frequently disrupt the normal operation of the system. In extreme scenarios, strategies learned from data may contravene knowledge rules, resulting in decision chaos or even system collapse, severely endangering driving safety and reliability.

**Figure 6:**
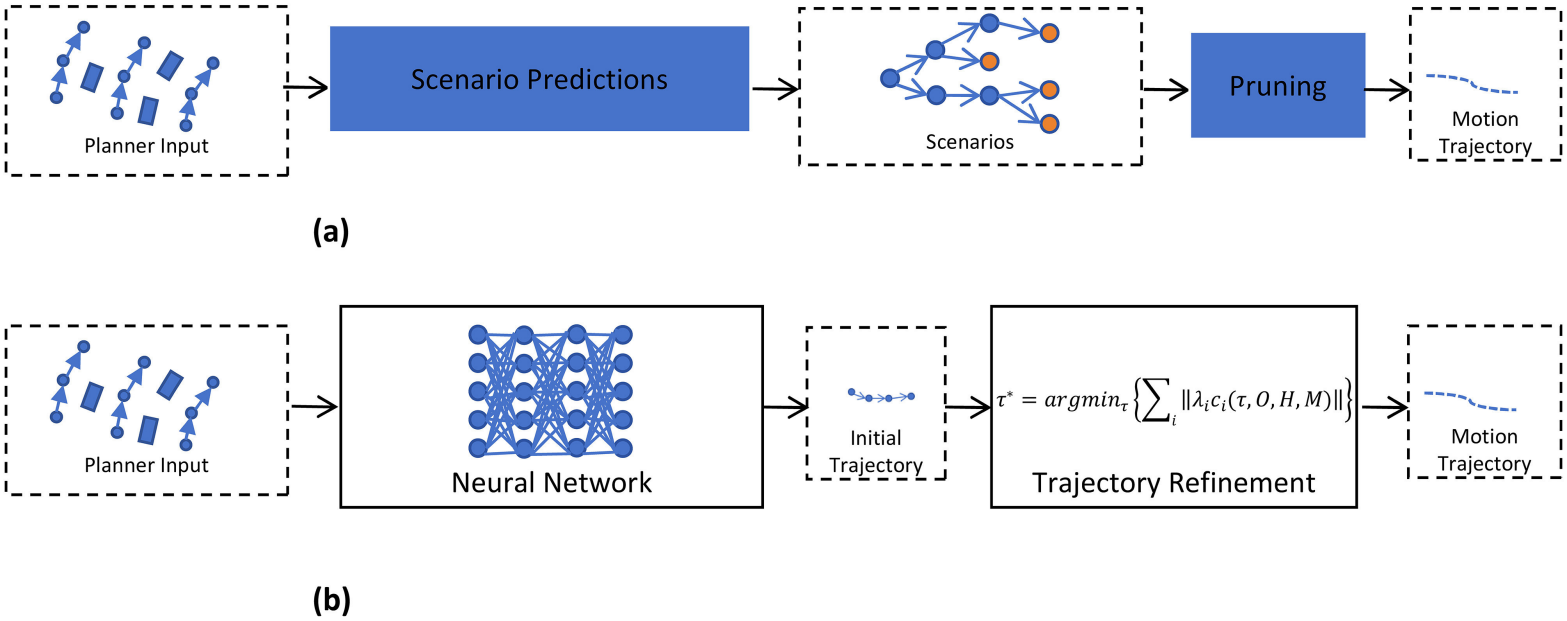
Two typical hybrid model pipelines. **(A)** Represents the pipeline of EPSILON, MARC, DTPP, TPP, and GameForme, and the **(B)** represents the pipeline of methods using neural networks to provide initial solutions.

The algorithmic framework of hybrid models achieves flexible and safe high-interactivity decision planning by integrating the data-driven learning capabilities with the interpretability, safety, and efficiency of knowledge-driven approaches. The knowledge-driven component of the hybrid model provides rules to ensure the legality, consistency, and interpretability of decisions. Simultaneously, the data-driven component offers insights to identify and adapt to complex scenarios and dynamic changes that fall outside the scope of predefined rules. Algorithms such as EPSILON (Ding et al., 2021), MARC (Li T. et al., 2023), DTPP (Huang et al., 2023a), TPP (Chen Y. et al., 2023), and GameFormer (Huang et al., 2023b) enhance the decision-making process through deep learning models, while using tree-based modeling to incorporate prior knowledge for rule constraints, the pipeline of these methods are shown in Figure 6A.

Specifically, EPSILON employs a two-tier structure consisting of a behavior planning layer and a motion planning layer. Initially, the algorithm generates a series of possible vehicle action sequences at the behavior planning layer using partially observable Markov decision process (POMDP) and branching guidance techniques. Neural network is employed to identify the potential intentions of surrounding vehicles. Subsequently, a scenario tree is constructed to simulate and evaluate traffic scenarios under different action sequences. During the multi-agent forward simulation phase, the algorithm assesses the potential risks and benefits of each scenario. Based on these assessments, it selects the optimal decision and converts it into specific trajectories with Spatiotemporal Semantic Corridors (SSC) to ensure the smoothness and safety of the trajectories. MARC utilizes a neural predictor to forecast the future trajectories and intentions of surrounding vehicles. It then goes through steps such as key vehicle selection, scenario tree construction, Risk-Aware Contingency Planning (RCP), and strategy evaluation and selection. MARC employs neural network-assisted risk assessment, enabling it to make decisions according to varying risk levels, thereby generating driving strategies that align with user risk preferences. DTPP leverages a Transformer model for conditional prediction and cost evaluation. The model uses the future trajectory branch information of the ego vehicle as the decoder's query vector, generating multi-stage predictions for the entire scenario tree and jointly optimizing them with the ego vehicle's planning. The TPP method transforms the continuous space motion planning problem into a discrete Markov decision process (MDP) by constructing ego vehicle trajectory trees and scenario trees and uses dynamic programming algorithms to find the optimal strategy. Neural networks play a crucial role in TPP by providing deep understanding and prediction of the traffic environment, thereby assisting the planner in making precise and efficient planning decisions in complex interactive environments. The GameFormer framework integrates hierarchical game theory and Transformer models, proposing a comprehensive solution for interaction prediction and planning of autonomous vehicles. This framework employs Long Short-Term Memory (LSTM) networks and Multi-Layer Perceptrons (MLP) to encode information, forming a scene context tensor that includes past features of all agents. Then the Transformer encoder captures relationships among all elements in the scene. The model adopts a hierarchical decoder structure where the level-0 decoder independently predicts future trajectories of agents, and the level-k decoder iteratively refines predictions based on the previous level's results. The self-attention mechanism is used to model interactions between future trajectories of agents, and the model parameters are optimized through end-to-end training using imitation loss and interaction loss.

Next, we will further elaborate on methods that utilize neural networks to provide initial solutions, the pipeline of these methods are shown in Figure 6B. SafetyNet (Vitelli et al., 2022) propose a system comprising a Machine Learning Planner (ML Planner) and a rule based Fallback Layer. The ML Planner generates trajectories by imitating human drivers' behavior, while the Fallback Layer performs plausibility checks, such as collision avoidance and ensuring physical feasibility. Liu et al. (2023) present a two-stage integrated neural planning framework that uses occupancy prediction to guide planning. Within this framework, Transformers and STrajNet are employed to generate initial planning trajectories. Hu et al. (2023a) a system is proposed that generates initial plans through a behavior cloning stage and optimizes trajectories with a trajectory refinement stage. In this work, ResNet is used as a CNN encoder to extract features, and a Unet structure integrates multi-scale features to capture detailed contextual information. Dauner et al. (2023) introduce three models: PDM-Open, PDM-Closed, and PDM-Hybrid. PDM-Open is a simple MLP used to predict future path points; PDM-Closed is based on the Model Predictive Control (MPC) model, combining prediction, proposal, simulation, scoring, and selection; PDM-Hybrid is a hybrid model that merges the advantages of open-loop prediction and closed-loop planning.

Additionally, some researchers try to leverage the strong exploration capabilities of reinforcement learning to enhance the flexibility of knowledge-driven methods. Goldsztejn and Brafman (2024) propose a method that combines classical planning algorithms with reinforcement learning. By using classical planners such as Dynamic Window Approach (DWA) to initialize the replay buffer of RL algorithms and employing an Actor-Critic method regularized by expert policies. This method also includes a supervisory module optimized with genetic algorithms, which dynamically switches to a safe policy when approaching obstacles, thereby enhancing the safety and adaptability of the strategy. Brito et al. (2021) present an interactive model predictive control (IntMPC) framework that integrates deep reinforcement learning with an optimization-based planner. The interaction-aware policies learned through DRL provide global guidance, assisting the optimization-based planner in generating control commands that adhere to dynamic and collision-avoidance constraints. Additionally, the paper introduces the Predictive Intelligent Driving Model (P-IDM) to simulate the driving behavior of other vehicles, with policies trained using the Soft Actor-Critic (SAC) algorithm. Zhang E. et al. (2023) propose a predictive trajectory planning framework based on reinforcement learning. This framework employs a partially observable Markov decision process (POMDP) model and a deep Q-learning solution to learn high-quality policies. It utilizes a Bayesian Gaussian Mixture Model (BGM) and Gibbs sampling to generate training data, and the DQN model is used to learn planning strategies from graph-represented traffic dynamics.

Hybrid models make an attempt to combine the learning capabilities of data-driven methods with the rule based logic of knowledge-driven approaches, aiming to achieve a better balance in autonomous driving decision-making and planning. However, this approach also faces some challenges, such as effectively integrating the two techniques, maintaining system stability and reliability, and addressing potential conflicts between data and rules. By exploring and comparing these different methods and their integrated applications, we can gain a more comprehensive understanding of the diversity and complexity that must be considered when designing advanced autonomous driving systems.

## 5 Experiment platform

The risks associated with testing system functionalities in real vehicle systems are unpredictable. Therefore, before conducting real-world road tests, autonomous driving algorithms are typically systematically evaluated using open-source datasets and simulation platforms.

### 5.1 Datasets

Autonomous driving simulation testing is a crucial step in verifying whether autonomous driving algorithms meet the expected standards. Therefore, the simulation testing process requires a large amount of data from autonomous driving scenarios. The stability, robustness, and generalization of the algorithm are directly influenced by the size of the dataset, the richness of the scenarios, and the distribution of the data. Hence, selecting an appropriate simulation platform and test data is of utmost importance.

The NuPlan (Caesar et al., 2021) dataset provides a large-scale and realistic driving dataset and evaluation framework specifically for the autonomous driving domain. This dataset encompasses 1500 hours of human driving data, covering multiple cities in the United States and Asia, which exhibit diverse traffic patterns and driving challenges. The dataset includes various sensor data such as LiDAR point clouds, camera images, localization information, and steering inputs. The nuPlan Challenge (Dauner et al., 2023), the world's first machine learning-based benchmark challenge for closed-loop planning for autonomous driving, provides a rich resource and evaluation framework for the field of autonomous driving planning research. It has a large-scale, high-quality human driving dataset from four cities in the US and Asia, providing semantic maps, sensor data, and automatically labeled agent trajectories, albeit with a large dataset size (200+TB) and only partial sensor data. Evaluation servers and templates allow users to submit ML-based planning codes for closed-loop evaluation, while the closed-loop simulation framework contains reactive agents for evaluating planning systems. In addition, the challenge provides generic and scenario-based planning metrics to comprehensively measure traffic rule compliance, human driving similarity, vehicle dynamics, and goal attainment.

One of NuPlan's significant innovations lies in its closed-loop evaluation framework, which allows containerized closed-loop evaluation of code on a confidential test set, ensuring the fairness and accuracy of the assessment process. Evaluation tasks are divided into open-loop and closed-loop challenges, requiring planning systems to simulate human driver behavior or control the vehicle in real-time. Evaluation metrics encompass traffic rule compliance, similarity to human driving, vehicle dynamics, goal achievement, and scenario-specific metrics. We selected the performance of several algorithms on the nuplan dataset. OL is open-loop data: trajectory planning is performed through playback, the planning result does not affect the vehicle, and the prediction result is compared with the empirical trajectory. CL-NR is closed-loop data without interaction: the planning result will directly act on the vehicle, but the surrounding vehicles will not be changed due to the self-vehicle's action. CL-R is closed-loop data with interaction: the planning results are directly applied to the vehicle, but the surrounding vehicles will change due to the actions of the self-vehicle. The results of the state-of-the-art method on nuplan are shown in Table 1.

**Table 1:** Advanced methods on nuplan.

**Method**	**OL**	**CL-NR**	**CL-R**
PDM (Dauner et al., 2023)	0.829	0.928	0.929
Hoplan (Hu et al., 2023a)	0.852	0.989	0.881
Muti-path (Xi et al., 2023)	0.876	0.817	0.851
Gameformer (Huang et al., 2023b)	0.840	0.809	0.838
PlanTF (Cheng et al., 2024b)	0.892	0.848	0.768
TPP (Chen Y. et al., 2023)	-	0.739	0.770
DTPP (Huang et al., 2023a)	0.791	0.896	0.898
Pluto (Cheng et al., 2024a)	-	0.932 (The paper does not clearly identify NR or R)	

The Waymo (Sun et al., 2020) Open Dataset is a large-scale and high-quality dataset containing LiDAR and camera data with detailed annotations including 2D and 3D bounding boxes. Its primary purpose is to support perception tasks for autonomous vehicles. The dataset provides researchers with rich resources to tackle real-world autonomous driving challenges, such as object detection and tracking. The high precision and diversity of the Waymo Open Dataset make it an important benchmark in the field of autonomous driving research. In the Waymo Open Sim Agents Challenge, the MVTE algorithm was selected for its closed-loop training execution, flexible and efficient strategy, and advanced and rational architecture. It is derived from the TrafficSim-inspired MVTA, based on the “backwards-looking” strategy, and improved by the MVTE. It is based on the TrafficSim-inspired MVTA, which has been improved by MVTE to enhance simulation diversity and accurately predict behaviors based on the transformer architecture and GMM header. The results of the state-of-the-art method on waymo are shown in Table 2 waymo Open Sim Agents Challenge (WOSAC) (Montali et al., 2024), WOSAC provides an autoregressive traffic agent-based evaluation framework that evaluates how well simulated agents match real-world sample distributions by approximating the negative log-likelihood (NLL). The evaluation platform and online rankings can be accessed via a URL for easy submission and viewing of rankings. In addition, the competition uses test data from Waymo Open Motion Dataset (WOMD) version v1.2.0, containing a large number of high-fidelity object behaviors and shapes with different data divisions. WOSAC provides evaluation criteria that provide a standardized way of evaluating simulated agents and help to compare the performance of different methods. By analysing the submitted methods, a number of trends were observed, and WOSAC is the first open challenge to address the task of simulating agents with realism and interactivity and to propose the corresponding metrics, filling a gap in benchmarks in the field. Through experiments and analyses, it was demonstrated that the learned, stochastic simulation agent outperforms the heuristic baseline and the learned, deterministic simulation agent in terms of combined metrics. In addition, a summary of the competition identifies the shortcomings of the existing dataset in terms of collision data and points out the direction of improvement for future work.

**Table 2:** Advanced methods on waymo.

**Agent policy**	**ADE**	**MINADE**
Wayformer (identical samples, 10 hz replan) (Nayakanti et al., 2023)	6.823	6.823
Sbta - adia (Mo et al., 2023)	4.777	3.611
Wayformer (identical samples, 2 hz replan) (Nayakanti et al., 2023)	2.498	2.498
CAD (kuang Chiu and Smith, 2023)	3.334	2.308
Wayformer (diverse samples, 2 hz replan) (Nayakanti et al., 2023)	2.588	1.694
Joint - multipath++^*^ (Varadarajan et al., 2022)	5.308	2.052
MRT+++ (Qian et al., 2023)	2.125	1.679
Mvta (Wang Y. et al., 2023)	3.938	1.870
Mvte (Wang Y. et al., 2023)	3.873	**1.677**

* Optimal version.

### 5.2 Simulation and testing tools

In 2023, the Waymo research team further expanded this data resource by developing the Waymax simulator, which matches the dataset. Waymax is a data-driven simulator that can use real-world driving data to initialize or recreate multi-agent simulation scenarios. This simulator is specifically designed for large-scale simulation and testing and supports hardware accelerators such as TPUs and GPUs to ensure efficient computational performance. This enables researchers to develop and evaluate autonomous driving planning software in a safe and cost-effective environment, significantly improving R&D efficiency and safety.

The Waymax simulator uses data from the Waymo Open Dataset to initialize simulation scenarios, creating a direct connection at the data level. This close integration allows Waymax to create highly realistic traffic environments and driving challenges based on real data, providing researchers with an authentic and challenging testing platform. This not only helps to validate the effectiveness and reliability of autonomous driving algorithms but also promotes the development and refinement of autonomous driving technology.

Testing autonomous driving algorithms in real-world environments often comes with numerous potential risks, and deploying these algorithms directly for real-world testing is both time-consuming and resource-intensive. A simulation testing environment provides a convenient and efficient way for validating intelligent algorithms.

Many autonomous driving simulation platforms are developed using open-source code and protocols, and they are extensively utilized for testing autonomous driving algorithms. Carla (Dosovitskiy et al., 2017) is an open-source simulation platform for autonomous driving, extensively utilized in the research and development of autonomous driving technologies. Developed collaboratively by Intel Labs and the Toyota Research Institute, this platform offers the industry a flexible and scalable simulation tool. Carla is built on the Unreal Engine 4, enabling the generation of highly realistic urban, suburban, and rural environments. This provides credible scenarios for the testing and validation of autonomous driving algorithms. Carla simulation platform boasts several key features. Firstly, its high-fidelity physics engine can accurately simulate vehicle dynamics, as well as various complex traffic conditions and weather scenarios. This allows researchers to test autonomous driving systems' performance in different environments within a safe and controlled virtual setting. Secondly, Carla supports the simulation of multiple sensors, including cameras, LiDAR, and radar, facilitating the integration and testing of multi-sensor fusion technologies. Additionally, Carla offers a rich set of API interfaces, supporting Python and C++ programming languages, making it convenient for developers to customize and extend the simulation environment. In practical applications. Carla is widely used across various research domains in autonomous driving. For instance, it plays a significant role in the development of perception systems by simulating real-world scenarios involving pedestrians, vehicles, and traffic signals, thereby enhancing the accuracy of object detection and recognition algorithms. Moreover, Carla is employed in path planning and decision-making research, where its realistic road and traffic environments are used to verify the effectiveness and safety of various planning algorithms. In the context of deep learning and reinforcement learning applications, Carla provides a vast amount of high-quality training data, promoting the advancement of data-driven methods in autonomous driving technology. The CARLA Autonomous Driving Ranking is designed to assess the driving proficiency of autonomous driving systems in real-world traffic scenarios, providing an open platform for fair, repeatable assessment and simplified comparisons. The task requires the system to face multiple traffic conditions through predefined routes containing different scenarios and weather conditions. Participation is divided into sensor and map modes, both of which can obtain advanced route descriptions, with sensor mode requesting limited sensor data and map mode also obtaining high-definition map data. Evaluation metrics are based on driving scores (the product of route completion and violation penalties), with penalties or treatments for violations and interruptions, and detailed information is recorded for each occurrence. The results of the state-of-the-art method on Carla are shown in Table 3.

**Table 3:** Advanced methods on carla.

**Team**	**Submission**	**Driving score**	**Route completion**
Interfuser	ReasonNet (Shao et al., 2023b)	79.95	89.89
Interfuser	InterFuser (Shao et al., 2023a)	76.18	88.23
PPX	TCP (Wu et al., 2022)	75.14	85.63
WOR	LAV (Chen and Krähenbühl, 2022)	61.85	94.46
DP	TransFuser (Prakash et al., 2021)	61.18	86.69
NFS	TCP reproduced (Wu et al., 2022)	58.56	83.14
DP	TransFuser (Prakash et al., 2021) (reproduced)	55.04	89.65

The Waymax (Gulino et al., 2024) Simulation Platform is a cutting-edge, multi-functional simulation platform extensively utilized in various fields such as industrial automation, robotic control, and intelligent systems development. This platform integrates high-performance computing capabilities with a flexible simulation environment, offering users an end-to-end simulation solution from design to validation. The Waymax Simulation Platform is characterized by its high scalability and flexibility. Users can customize simulation models and parameter settings according to specific needs, thereby accommodating different types of simulation tasks. Additionally, the platform supports various simulation modes, including real-time simulation, distributed simulation, and hybrid simulation. The Waymax Simulation Platform also features robust data analysis and visualization tools. Through these tools, users can monitor the simulation process in real-time, analyze simulation results, and generate detailed reports and charts. These functionalities not only enhance the efficiency of simulation work but also provide users with means to gain deep insights and optimize system performance. The other simulation platforms and their related descriptions are shown in Table 4.

**Table 4:** Autonomous driving simulation platform and related descriptions.

**Platform**	**Open source licenses**	**Latest version**	**Language**
Carla (Dosovitskiy et al., 2017)	MIT License	V0.9.15	Python, C++
Waymax (Gulino et al., 2024)	Apadhe 2.0	V2023	Python
Autoware (Miura et al., 2019)	Apache 2.0	V1.14.0	C++, Python
Apollo (Xu et al., 2019)	Apache 2.0	V9.0	C++
MetaDrive (Li et al., 2022)	Apache 2.0	V0.4.2.3	Python
LGSVL (Rong et al., 2020)	Apache 2.0	V2023.1	C#, Python
TESS-NG (Kang et al., 2020)	Closed source	V3.3	Qt, C++
CarMaker (Ren et al., 2021)	Closed source	V13.0	C++

## 6 Challenges and future perspectives

### 6.1 Current challenges in decision and planning

A. Uncertainty in environmental perception significantly impacts decision-making and planning systems in autonomous driving. Sensor limitations and data fusion issues can lead to misinterpretations of the environment, affecting the system's judgments and choices. Additionally, dynamic and complex environmental factors, such as changing traffic flows and sudden events, require high adaptability and real-time response. Increased uncertainty complicates the prediction and handling of these situations, potentially leading to unstable decisions. Therefore, advancements in sensor technology, data processing, model optimization, and algorithm innovation are essential to improve the safety and reliability of autonomous driving systems.

B. Another major challenge in autonomous driving is handling the uncertainty of other traffic participants' behaviors. Actions of vehicles, pedestrians, and cyclists are highly unpredictable, requiring real-time monitoring and prediction. This complexity demands advanced perception capabilities and sophisticated algorithms for behavior prediction, involving extensive data processing and machine learning. Additionally, the system must make conservative decisions to ensure safety when predictions are uncertain. Thus, addressing this unpredictability requires advanced perception, complex prediction algorithms and conservative safety strategies.

C. The application of data-driven algorithms in autonomous driving faces significant challenges related to data comprehensiveness and generalization. First, data comprehensiveness is critical as effective decision-making requires understanding diverse road conditions, traffic behaviors, and rare events. However, collecting and labeling datasets that cover all potential scenarios is extremely difficult, leading to gaps that can cause models to perform poorly in unseen situations. Second, generalization is a concern as algorithms may not remain effective in new environments not represented in the training data. This can result in errors or failures to adapt, posing safety risks, such as misinterpreting traffic rules in different regions.

D. The lack of interpretability in data-driven algorithms poses major challenges for autonomous driving decision-making. Firstly, safe operation relies on reliable and precise decisions, and interpretability is key to building user and public trust. If users can't understand the vehicle's behavior or logic, distrust may arise, affecting social acceptance and adoption. Secondly, in the event of abnormal behavior or accidents, poor interpretability complicates investigations and accountability, increasing legal and ethical risks and potentially leading to stricter regulations. Additionally, non-interpretable decision processes hinder debugging and optimization, as developers struggle to diagnose and fix issues, leading to system instability and unpredictable behavior. This also limits further algorithm development and fine-tuning.

### 6.2 Future perspectives

**Enhancing perception and reducing uncertainty:** Enhancing perception and reducing uncertainty in autonomous driving can be achieved through multi-sensor fusion and advanced deep learning techniques. By integrating data from various sensors such as cameras, radar, and LiDAR, a more comprehensive understanding of the environment is possible. This fusion technology offers stable and reliable perception under diverse conditions (e.g., fog, night), mitigating the limitations and errors of individual sensors. Additionally, deep learning and artificial intelligence enhance the understanding of dynamic and complex environments. These models learn high-level features from vast datasets, improving decision-making and judgment in uncertain situations.

**Improving prediction of other traffic participants behavior:** Improving the prediction of other traffic participants' behavior involves developing advanced behavior prediction models and utilizing simulation technologies. Machine learning-based predictive models can simulate and understand interactions among traffic participants, forecasting the possible actions of vehicles and pedestrians in the coming seconds to inform safer driving strategies. Additionally, creating virtual driving environments to simulate complex traffic scenarios helps algorithms learn to respond under unknown or extreme conditions. Augmented reality technology further enhances this by simulating potential hazards during actual driving, thereby improving the system's response capabilities.

**Enhancing data comprehensiveness and generalization:** Enhancing data comprehensiveness and generalization involves generating synthetic data to train models for rare or unseen scenarios and using domain adaptation techniques to minimize performance variability across different geographic and environmental conditions. Additionally, implementing online learning capabilities in algorithms allows them to update and optimize in real time based on newly collected data, thereby adapting to continuously changing environments and conditions.

**Improving algorithm interpretability:** Improving algorithm interpretability involves developing interpretability mechanisms, such as visualization techniques or generating explanatory texts, to help users understand the rationale behind model decisions. Additionally, combining traditional rule based systems with modern deep learning methods not only maintains decision efficiency but also increases transparency and traceability in the decision-making process.

**Drawing on neuroscience to enhance the intelligence of foundational models:** In recent years, interdisciplinary contributions from psychology and neuroscience have increasingly influenced the development of self-driving vehicles. Insights from neuroscience, particularly in understanding human perception, decision-making, and sensorimotor integration, have inspired advancements in algorithms for autonomous systems. For example, studies on how humans process visual and auditory cues in dynamic environments have informed the design of multi-sensory data fusion techniques in self-driving cars. Similarly, psychological research on human behavior, attention, and cognitive biases has contributed to the development of more intuitive human-machine interfaces and predictive models that anticipate pedestrian and driver actions. By integrating these principles, the field of self-driving vehicles not only achieves more robust performance but also fosters safer and more human-centric designs. Future work could benefit further from deeper collaborations across these disciplines, particularly as neurorobotics continues to bridge the gap between biological and artificial systems. In the future, psychology and neuroscience are expected to play a crucial role in advancing human-centric and intelligent self technologies. Neuroscience research on human perception and decision-making can inspire innovative algorithms by offering insights into how the brain processes multimodal information in dynamic environments, improving sensor data fusion and real-time decision-making. Similarly, psychological studies on human behavior and cognitive processes will enhance the safety and naturalness of human-machine interactions, helping design interfaces that align with human habits and reduce risks in interactions with passengers and road users. Furthermore, as neurorobotics evolves, self-driving systems may achieve greater adaptability and human-like intelligence by emulating the functioning of biological brains and neural networks. Realizing this vision will require interdisciplinary collaboration across psychology, neuroscience, artificial intelligence, and engineering, fostering safer, more efficient, and human-centric autonomous technologies while exploring the relationship between artificial and human intelligence.
